# Supplementary material for: Cdc42 coordinates apical membrane formation and secretory granule maturation through v-SNARE maintenance in salivary acinar cells
Source: J Biol Chem. 2026 Sep 7;302(10):113521. doi: 10.1016/j.jbc.2026.113521 (PMC13661217; doi:10.1016/j.jbc.2026.113521)
Supplement: Supplementary file 1 [file mmc3.pdf]

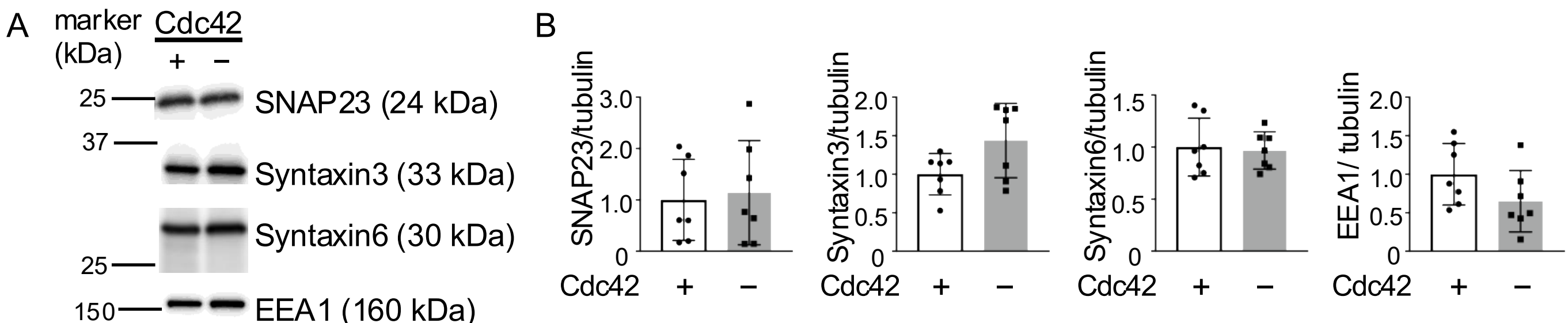
A
marker (kDa)
Cdc42
+ −
25
37
25
150
SNAP23 (24 kDa)
Syntaxin3 (33 kDa)
Syntaxin6 (30 kDa)
EEA1 (160 kDa)
B
SNAP23/tubulin
Syntaxin3/tubulin
Syntaxin6/tubulin
EEA1/ tubulin
Cdc42 + −
