# Supplementary material for: Cdc42 coordinates apical membrane formation and secretory granule maturation through v-SNARE maintenance in salivary acinar cells
Source: J Biol Chem. 2026 Sep 7;302(10):113521. doi: 10.1016/j.jbc.2026.113521 (PMC13661217; doi:10.1016/j.jbc.2026.113521)
Supplement: Supplementary file 2 [file mmc4.pdf]

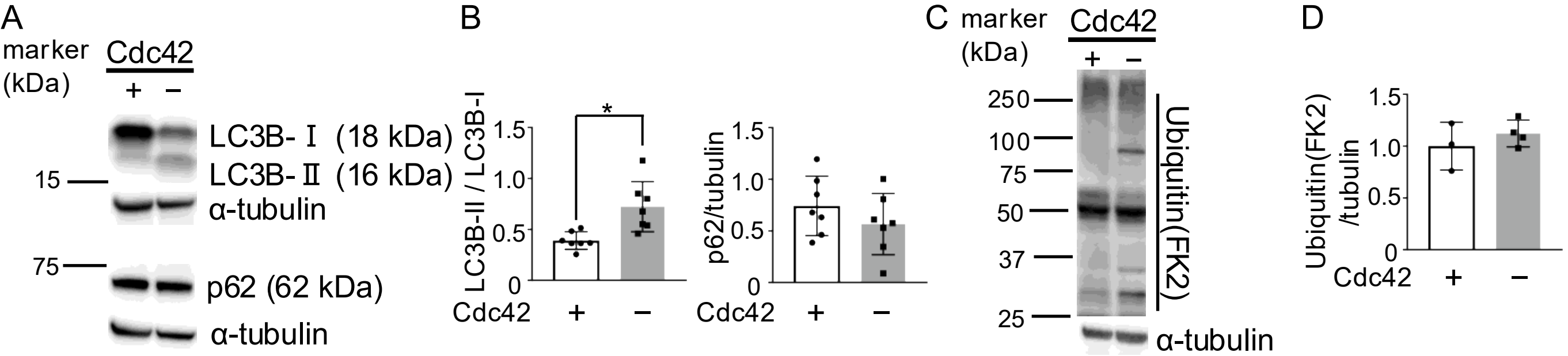

A
marker (kDa)
Cdc42
+ –
LC3B- Ⅰ (18 kDa)
LC3B- Ⅱ (16 kDa)
15
α-tubulin
75
p62 (62 kDa)
α-tubulin
B
LC3B-II / LC3B-I
1.5
1.0
0.5
0
*
Cdc42 + –
p62/tubulin
1.5
1.0
0.5
0
Cdc42 + –
C
marker (kDa)
Cdc42
+ –
250
100
75
50
37
25
Ubiquitin(FK2)
α-tubulin
D
Ubiquitin(FK2) /tubulin
1.5
1.0
0.5
0
Cdc42 + –
