# Supplementary material for: Cdc42 coordinates apical membrane formation and secretory granule maturation through v-SNARE maintenance in salivary acinar cells
Source: J Biol Chem. 2026 Sep 7;302(10):113521. doi: 10.1016/j.jbc.2026.113521 (PMC13661217; doi:10.1016/j.jbc.2026.113521)
Supplement: Supplementary file 3 [file mmc1.docx]

**Supplementary Figure S1. Trafficking, endocytic, and cytoskeletal proteins are unchanged in Cdc42-deficient glands.**

(A) Western blot analysis of the t-SNAREs SNAP23, Syntaxin3, and Syntaxin6; and the endosomal marker EEA1 in parotid glands from control (Cdc42⁺) and Cdc42⁻ mice. Representative blots; molecular weights are indicated.

(B) Quantification normalized to α-tubulin (mean ± S.D.). t-SNAREs: SNAP23 (unchanged, P = 0.78; n = 7 per group), Syntaxin3 (1.4-fold, not significant, P = 0.058; n = 7 per group), Syntaxin6 (unchanged, P = 0.80; n = 7 per group). Endosomal marker: EEA1 (unchanged; n = 7 per group). None of these proteins were significantly reduced, in contrast to the selective decrease of VAMP2 and VAMP4 (Figure 3), indicating that Cdc42 deficiency does not broadly lower the fusion machinery, or endocytic proteins but selectively reduces a specific subset of v-SNAREs.

**Supplementary Figure S2. Ubiquitination, LC3B, and p62 in Cdc42-deficient glands.**

(A) Western blot analysis of LC3B (LC3B-I, 18 kDa; LC3B-II, 16 kDa) and p62 (62 kDa) in parotid glands from control (Cdc42⁺) and Cdc42⁻ mice, with α-tubulin as a loading control.

(B) Quantification of the LC3B-II/LC3B-I ratio and of p62 normalized to α-tubulin (mean ± S.D.). The LC3B-II/LC3B-I ratio was increased in Cdc42⁻ glands (Cont., 0.39 ± 0.09; KO, 0.72 ± 0.25; n = 7 per group; *P = 0.011, Welch's t-test), whereas p62 was unchanged (Cont., 0.74 ± 0.29; KO, 0.57 ± 0.30; n = 7 per group; P = 0.28, unpaired t-test).

(C) Western blot analysis of ubiquitinated proteins (FK2) in parotid glands from control (Cdc42⁺) and Cdc42⁻ mice, with α-tubulin as a loading control.

(D) Quantification of ubiquitinated proteins (FK2), measured as the total signal across the 30–250 kDa range of each lane and normalized to α-tubulin (mean ± S.D.). Ubiquitination was unchanged (Cont., 1.00 ± 0.23; KO, 1.12 ± 0.13; n = 3 control, 4 knockout; P = 0.41, unpaired t-test). One control sample (WT3) was excluded because of markedly reduced α-tubulin signal (see Methods).
